# Leader Affective Presence and Feedback in Teams

**DOI:** 10.3389/fpsyg.2020.00705

**Published:** 2020-05-05

**Authors:** Hector P. Madrid

**Affiliations:** Pontifical Catholic University of Chile, Santiago, Chile

**Keywords:** affective presence, feedback, leaders, teams, Leadership

## Abstract

Affective Presence refers to the consistent and stable feelings that an individual tends to leave in their interaction partners. Expanding previous research on the application of affective presence to individuals in the role of leaders in teams, in this study, I examine whether leaders’ feedback behavior is related to the emergence of their affective presence. Using a multisource survey design with a sample of employees from a technology organization, as expected, results indicated that leader feedback behavior directed to the characteristics that team members should ideally have to perform well (ought self feedback) is positively related to leaders’ positive affective presence. In contrast, feedback directed to compare team members’ performance with other team members’ performance (normative cues feedback) is positively associated with leaders’ negative affective presence. As such, the study contributes to having an understanding of the etiology of affective presence in the context of teams, also informing organizational practitioners about how to manage leader influences on team members.

When interacting with others, Paul tends to provoke enthusiasm in his interaction partners, while Julia is prone to make their interaction partners feel anxious. If these effects are consistent over time and independent from the situation, researchers have labeled them as *affective presence*. Formally, affective presence refers to the consistent and stable feelings that a person tends to provoke with their interaction partners, which is a complementary psychological process to emotional contagion (Eisenkraft and Elfenbein, 2010). While contagion is the propagation of an individual’s inner-life feelings toward interaction partners, affective presence is the individual’s elicitation of feelings in others, separate from how the individual actually feels. Applying to the work setting in organizations, previous research has shown that affective presence of team leaders has the potential to benefit or dampen team member collaboration, information sharing, creativity, innovation and service performance, due to the functions of affect that shape cognition and behavior in the interpersonal realm (Madrid et al., 2016; Jiang et al., 2018). Thus, research on affective presence is offering a new avenue to understand and manage leaders’ affective influence on their followers. However, our knowledge regarding where the individuals’ affective presence stems from is still scant and limited (Berrios et al., 2014), and represents a significant gap, the exploration of which would allow for a more comprehensive understanding of what this construct is, as well as its psychological roots.

In emerging studies, affective presence has been defined as a personality trait, in that the tendency to provoke feelings in others should be the cause of subsequent behavior in the interpersonal domain (Eisenkraft and Elfenbein, 2010; Madrid et al., 2018). However, this conceptualization is debatable because personality traits involve individual tendencies in cognition, emotion and behavior, which are conditions not satisfied by the affective presence phenomenon. Alternatively, I argue that affective presence is part of the personality system, but it denotes an affective expression of psychological traits and recurrent behavioral patterns. As such, individuals’ behavior should be the cause, but not the result, of the stable way that they make others feel. Based on the above, in this study, I examine whether team leaders’ behavior explains the emergence of their affective presence, with a focus on the use of feedback strategies directed to the members of their teams. Specifically, I argue and test whether leader feedback behaviors directing the attention of team members to their self or directing the attention to performance of others are related to leaders’ positive and negative affective presence, respectively.

Leaders’ feedback behavior is a critical element in organizational life because it helps to meet the performance standards expected, based on the provision of information to team members about their past performance and, therefore, reinforce or modify work-related behavior (Aguinis et al., 2012). Feedback behavior is multidimensional, such that when focused on employee behavior, feedback can be directed to the self of the employee highlighting the characteristics that he or she should have to perform well (ought self feedback). In turn, feedback could also be directed to compare the performance of the employee with that shown by other employees (normative cues feedback) (Kluger and DeNisi, 1998).

In general, leaders’ behavior may participate in the construction of their affective presence due to the informational meaning of affect in the interpersonal realm. According to theories about affect in the social context (Parkinson, 1996; Van Kleef, 2009), feelings given in social interactions communicate the goals, intentions, motivations, and attitudes of a focal person toward their interaction partners and the relationship among them, such as the case of the relationship between leaders and their followers (Van Knippenberg and Van Kleef, 2016). Thus, in general, positive affect conveys positive attitudes about the social interaction and consideration for interaction partners, whereas the opposite applies to negative affect (Van Kleef et al., 2012).

Based on the above, leader affective presence in the context of teams may emerge from the delivery of feedback because of the interpersonal implications of this behavior, which would denote the attitudes and consideration of leaders for their team members. In the case of feedback directed to the ought self, team members’ selves are directly taken into account, and thereby they might infer that the leader is concerned and considerate of them, which should be linked to positive affect (cf., Higgins, 1997). On the other hand, feedback based on normative cues, in which case the leader performs social comparisons between followers, may cause team members to feel their selves threatened, and therefore feel uncomfortable, which would be denoted as a lack of concern from leaders toward them. This kind of psychological process is often related to negative affect (Kluger and DeNisi, 1998; Brockner and Higgins, 2001). Accordingly, theory and evidence about feedback interventions suggest that feedback behavior directed to the ought self has the potential to lead to positive outcomes, while feedback directed to normative cues is an ineffective strategy. Drawing on this, I propose that team leaders’ feedback behavior directed to team members’ ought self would be positively related to their positive affective presence, while leaders’ feedback oriented to normative cues would be positively associated with their negative affective presence.

Hypothesis 1: Leader feedback directed to the ought self will be positively related to leader positive affective presence.
Hypothesis 2: Leader feedback directed to normative cues will be positively related to leader negative affective presence.

## Method

To test the above hypotheses, I conducted a multisource survey study with employees from a technology organization. In this, leaders of different working teams answered a survey asking them about the feedback strategies they delivered with members of their teams, while an independent survey was conducted with team members to measure the affective presence of their leaders, together with leader-member interaction frequency and team members’ extraversion and neuroticism to be used as control variables. Twenty-six leaders and 143 team members participated in the study. The gender split among leaders was 73% males, their average age was 38.23 years (*SD* = 7.8), and their average organizational tenure was 4.06 years (*SD* = 1.14). Team members were 71% male; their average age was 30.62 years (*SD* = 8.17) and their average organizational tenure 3.9 years (*SD* = 4.29). Teams performed executive, management, production, and service functions (Devine, 2002), associated with finance and administration, human resource management, information systems, marketing and sales, logistics and distribution, retail, customer service, and technical support. Average team size was 5.5 team members (*SD* = 1.03, Min. = 2, Max. = 3), and average intra-team response rate was 59%.

Feedback behavior was measured with six items developed for the study. Leaders were asked about a series of statements about their feedback behavior directed to the team members’ ought self as a whole (2 items: “I make team members think about how they should ideally be at work,” “I make team members think about the skills they need to perform well at work,” *r* = 0.65) and directed to normative cues (2 items: “I compare team members with other team members’ performance,” “I use other members as an example of performance,” *r* = 0.67). According to theory on feedback, performance information delivered can also involve how well the tasks are performed in general, independent from how well employees are conducting their work behavior (Kluger and DeNisi, 1996). Thus, a measure with 2 additional items were utilized as covariate in the model to control possible confounding effects associated with this feedback strategy on affective presence (“I focus team members attention on the tasks they have to perform,” “I make team members pay attention to the tasks expected from them,” *r* = 0.60). Leader affective presence was measured with the scale developed by Madrid et al. (2016), in which team members were asked about to what extent when interacting with their leaders they make them feel “enthusiastic,” “joyful” and “inspired” (positive affective presence, α = 0.90), and “anxious,” “worried” and “tense”) (negative affective presence, α = 0.79). Team members’ extraversion and neuroticism were measured with 8 items from the scale developed by Benet-Martínez and John (1998), in which they indicate their agreement with a series of statements, such as “I am a person who…” “generates a lot of enthusiasm” (extraversion, α = 0.82) and “gets nervous easily” (neuroticism, α = 0.71). These personality traits were included in the study to account for possible systematic influences of affective components of the team members’ personality (i.e., enthusiasm, anxiety) on their reports about leader affective presence. In turn, following previous research (Madrid et al., 2018), leader-member interaction frequency was measured with a single item, which asked team members the extent to which they interact with their leaders (1: almost never – 5: every day). This measure was useful to control possible effects of team members’ exposure intensity to leader behavior on perceptions of leaders’ affective presence.

The data analysis strategy consisted of confirmatory factor analysis to determine the robustness of the measurement model (Brown, 2006). Furthermore, inter-rater agreement analysis was conducted to examine whether team members’ ratings of leader affective presence were non-independent relative to their leaders and if team members shared the same feelings elicited by leaders (LeBreton and Senter, 2008). Finally, hypotheses were tested using two-level random intercept models, in which leader affective presence variance was partitioned into within- and between-subjects components at levels 1 and 2, respectively, to account for the nested structure of data around leaders (Hox, 2010). Team members’ interaction frequency with their leaders and their extraversion and neuroticism were defined as level-1 predictors, whereas task, ought self, and normative cues feedback as level-2 predictors.

Data were analyzed with *R Statistical Package*. Results of confirmatory factor analysis supported the robustness of the measurement model described by all the variables measured in the study [χ^2^(*df*) = 251.08(151), *p* < 0.05; RMSEA = 0.07, CFI = 0.92]. Furthermore, inter-rater reliability and agreement analyses supported that team members’ ratings of leader affective presence were nested around their respective leaders and they showed strong inter-rater agreement (ICC1 = 0.07–0.30; AD = 0.78–0.89). Descriptive, correlations and reliabilities results are presented in Table 1. Results of the two-level random intercepts model (Table 2, Model 1) showed that feedback behavior directed to the ought self were positively related to leader positive affective presence (*b* = 0.37, *SE* = 0.15, *p* < 0.05), while feedback strategies directed to the normative cues were positively related to leader negative affective presence (*b* = 0.20, *SE* = 0.09, *p* < 0.05) (Table 2, Model 2). Therefore, hypothesis 1 and 2 were supported. Furthermore, although not anticipated, feedback strategies directed to the ought self were negatively related to leader negative affective presence (*b* = −0.11, *SE* = 0.09, *p* < 0.05) and feedback behavior directed to the normative cues were negatively related to positive affective presence (*b* = −0.42, *SE* = 0.13, *p* < 0.01).

**TABLE 1:** Means, standard deviations, correlations and reliabilities.

**Variables**	**Mean**	***SD***	**1**	**2**	**3**	**4**	**5**	**6**	**7**	**8**
1. Interaction frequency	4.06	0.63	–							
2. Extraversion	4.12	0.29	0.05	**(0.82)**						
3. Neuroticism	2.81	0.38	–0.12	–0.12	**(0.71)**					
4. Feedback directed to tasks	4.01	0.66	–0.10	–0.01	–0.13	–				
5. Feedback directed to the ought self	3.97	0.71	0.00	–0.09	–0.13	0.44**	–			
6. Feedback directed to normative cues	2.23	0.79	–0.12	–0.01	0.12	0.20**	0.13	–		
7. Positive affective presence	3.20	0.71	0.47**	0.17*	−0.36**	–0.09	0.15	−0.34**	**(0.90)**	
8. Negative affective presence	2.46	0.44	−0.39**	–0.04	0.37**	0.10	–0.06	0.21*	−0.56**	**(0.79)**

N = 26. Reliabilities are in bold and displayed in parentheses in the diagonal. * p < 0.05, ** p < 0.01.

**TABLE 2:** Multilevel modeling.

**Variables**	**Model 1 positive AP**	**Model 2 negative AP**
**Level 1**		
Interaction frequency	0.41(0.07)**	−0.31(0.07)**
Extraversion	0.23(0.10)*	
Neuroticism		0.39(0.10)**
**Level 2**		
Feedback directed to tasks	−0.17(0.17)	0.18 (0.10)
Feedback directed to the ought self	0.37(0.15)*	−0.11(0.09)*
Feedback directed to normative cues	−0.42(0.13)**	0.20(0.09)*
Deviance (-2 loglikelihood)	1271.94	1258.40

N = 140/26. Unstandardized estimates, standard errors are in parenthesis. * p < 0.05, ** p < 0.01.

## Discussion

This study supported that leaders’ feedback behavior participates in the construction of their affective presence in teams. As expected, feedback directed to the ought self was positively related to leader positive affective presence, while feedback directed to normative cues was positively related to leader negative affective presence. Although not hypothesized, asymmetrical effects were also observed, in that feedback directed to the ought self was negatively related to negative leader negative affective presence. In contrast, feedback directed to normative cues was negatively related to leader positive affective presence. I argue that such relationships are possible due to the informational meaning of leader behavior relative to their attitudes toward the relationship with team members (Van Kleef, 2009; Van Knippenberg and Van Kleef, 2016). Positive affective presence is associated with leaders’ positive attitudes toward the relationship and focus on the development of team members’ characteristics for achieving positive performance, but the opposite applies to negative affective presence.

These results contribute to expanding theory and evidence underlying the phenomenon of leader affective presence because etiology factors that explain the emergence of this construct in the interpersonal realm are still underdeveloped. Also, the findings observed here inform practical interventions for leader development. Since leader affective presence is associated with feedback behavior, training on the delivery of information to benefit team members’ performance should be a valuable strategy to foster functional teamwork, due to the benefits of affective presence for collaboration and creative, innovative, and service performance (Madrid et al., 2016; Jiang et al., 2018).

Limitations of the study involve the use of a cross-sectional survey design that might introduce issues on common method bias (Podsakoff et al., 2012). Although these possible problems were controlled by the use of a multisource strategy in the data collection and the inclusion of team members’ extraversion and neuroticism as control variables (Spector, 1994), issues about method variance might still be present in the statistical estimations performed. Furthermore, the same design was not suitable to establish a definitive causal order between the variables studied. Here, affective presence was assumed as a construct driven by behavioral tendencies of leaders expressed in feedback strategies; thus, affective presence would be a result of the individual’s behavior. However, other researchers have defined affective presence as a personality trait (Eisenkraft and Elfenbein, 2010), namely, a stable psychological tendency that shapes subsequent behavior, in which case leader feedback behavior might be the resulting medium by which leader affective presence is manifested (Madrid et al., 2018). Because theoretical and empirical research on affective presence is based only on a handful of studies, the conceptualization of this construct is still a matter for discussion, which stresses the relevance of conducting further research utilizing experimental and longitudinal studies. Another limitation was the sample size utilized in the study. Although a large number of team members participated in the study, which provides reliability for the ratings of affective presence, the number of team leaders was limited. Thus, the implementation of additional studies with larger sample sizes will be informative about whether the results observed here are robust and replicable.

Finally, future research should examine possible mediators between leaders’ behavior and their affective presence, other than inferences about leaders’ attitudes toward the social relationship. For example, as part of interpersonal processes, attributions of warmth and competence toward leaders might be a vehicle for the relationship between leaders’ behavior and the way they make their team members feel (Eisenkraft and Elfenbein, 2010; Cuddy et al., 2011). For example, in the context of this study, leader feedback behavior directed to the ought self might lead team members to perceive that the leader is concerned with their team members (warmth) and skillful in providing performance information (competent). Another opportunity for further research is the examination of leader affective presence strength (Madrid et al., 2018). The conceptualization of affective presence adopted here, as the same as in previous studies, is based on the level of agreement among team members about the extent to which leaders make them feel consistent positive or negative feelings. In contrast, leader affective presence strength refers to the extent to which the leader elicits the same feelings among team members, but with different intensity depending on the team member. In this case, affective presence is denoted by disagreement about the affective experience elicited among team members. Accordingly, affective presence strength might be explained because the leader behaves in a different way toward team members, such as using different feedback strategies with each of them. Another case is when the leader utilizes LMX strategies by delivering different degrees of autonomy and trust, depending on the team member. These issues highlight interesting opportunities to expand our understanding of the affective presence construct.

To sum up, this is an initial effort to uncover the etiology of affective presence in the context of teamwork. I trust that evidence built here will contribute to the literature and practice about leader affective influences in organizations.

## Data Availability Statement

The datasets generated for this study are available on request to the corresponding author.

## Ethics Statement

The studies involving human participants were reviewed and approved by the Ethics committee Pontificia Universidad Católica de Chile. The patients/participants provided their written informed consent to participate in this study.

## Author Contributions

HM: research problem, literature review, hypotheses elaboration, study design, data collection, data analysis and manuscript writing.

## Conflict of Interest

The authors declare that the research was conducted in the absence of any commercial or financial relationships that could be construed as a potential conflict of interest.
